# Circadian gene ARNTL initiates circGUCY1A2 transcription to suppress non-small cell lung cancer progression via miR-200c-3p/PTEN signaling

**DOI:** 10.1186/s13046-023-02791-1

**Published:** 2023-09-04

**Authors:** Deze Zhao, Yeping Dong, Minghao Duan, Dan He, Qun Xie, Wei Peng, Weifang Cui, Junjie Jiang, Yuanda Cheng, Heng Zhang, Faqing Tang, Chunfang Zhang, Yang Gao, Chaojun Duan

**Affiliations:** 1 Department of Thoracic Surgery, Xiangya Hospital, Central South University, Changsha, 410008 Hunan China; 2 Hunan Engineering Research Center for Pulmonary Nodules Precise Diagnosis & Treatment, Changsha, 410008 Hunan China; 3 Shulan International Medical College, Zhejiang Shuren University, Hangzhou, Zhejiang, 310011 China; 4 Hunan Key Laboratory of Oncotarget Gene, Hunan Cancer Hospital & The Affiliated Cancer Hospital of Xiangya School of Medicine, Central South University, Changsha, 410008 Hunan China; 5 Department of Ultrasonic Imaging, Affiliated Hospital of Hunan Traditional Chinese Medicine Research Institute, Changsha, 410006 Hunan China; 6 Department of Oncology, Hunan Provincial People’s Hospital, the First Affiliated Hospital of Hunan Normal University, Changsha, 410006 Hunan China; 7 Xiangya Lung Cancer Center, Xiangya Hospital, Central South University, Changsha, 410008 Hunan China; 8 Institute of Medical Sciences, Xiangya Hospital, Central South University, Changsha, 410008 Hunan China; 9 National Clinical Research Center for Geriatric Disorders, Changsha, 410008 Hunan China

**Keywords:** Non-small cell lung cancer, circGUCY1A2, miR-200c-3p, PTEN, ARNTL

## Abstract

**Background:**

As a subclass of endogenous stable noncoding RNAs, circular RNAs are beginning to be appreciated for their potential as tumor therapeutics. However, the functions and mechanisms by which circRNAs exert protective functions in non-small cell lung cancer (NSCLC) remain largely elusive.

**Methods:**

The prognostic role of circGUCY1A2 was explored in lung adenocarcinoma specimens. The overexpressed and knockdown plasmids were used to evaluate the effect of circGUCY1A2 on NSCLC cell proliferation and apoptosis efficacy. Luciferase reporter system is used to prove that circGUCY1A2 could bind to miRNA. Chip-PCR was used to prove that circGUCY1A2 could be initiated by transcription factors ARNTL. Subcutaneous tumorigenicity grafts models were established to validate findings in vivo.

**Results:**

The expression of circGUCY1A2 were significantly reduced (*P* < 0.001) and negatively correlated with tumor size (*P* < 0.05) in non-small cell lung cancer (NSCLC). CircGUCY1A2 upregulation promoted apoptosis and inhibits cell proliferation and growth of subcutaneous tumorigenicity grafts in nude mice (P < 0.01). In addition, intra-tumor injection of pLCDH-circGUCY1A2 inhibited tumor growth in patient-derived NSCLC xenograft models (PDX). Mechanism studies showed that circGUCY1A2 could act as a sponge to competitively bind miR-200c-3p, promote PTEN expression, and thereby inhibit PI3K/AKT pathway. In addition, we found that the circadian gene ARNTL, which was reduced in NSCLC and prolonged the overall survival of patients, could bind to the promoter of circGUCY1A2, thereby increasing its expression.

**Conclusions:**

This study is an original demonstration that ARNTL can inhibit the development of lung adenocarcinoma through the circGUCY1A2/miR-200c-3p/PTEN axis, and this finding provides potential targets and therapeutic approaches for the treatment of lung adenocarcinoma.

**Supplementary Information:**

The online version contains supplementary material available at 10.1186/s13046-023-02791-1.

## Background

Currently, the cancer profile in China is undergoing a shift and convergence to the United States, especially with an aging population exacerbating the social oncology burden [1]. In 2023, lung cancer will remain the leading cause of cancer death in both countries [1, 2]. However, due to lags in prevention and care, most lung cancer patients in China are diagnosed at advanced stages, thus leading to a poor prognosis [3, 4]. At present, the treatment strategies for lung cancer are developing rapidly, and there are breakthroughs but also limitations [5]. In-depth understanding of the pathogenesis of lung cancer and finding effective biomarkers have always been urgent problems in the field of oncology. Several studies have shown that circRNA dysregulation was implicated in progression of NSCLC. Their detectability and relative stability in body fluids make them potential cancer biomarker candidates [6–8].

CircRNA is an endogenous non-coding RNA, which is mainly produced by joining the downstream 3’-terminal and upstream 5’-terminal through direct back-splicing or a lariat driven back-splicing [9]. The common biological functions of circRNAs can be divided into four types: (1) miRNA sponges, (2) binding promoters and translation initiation proteins to regulate transcription and translation, (3) participating in protein interactions as scaffolds, and (4) possessing open reading frames to translated into peptides or proteins. Current studies have found that circRNA is involved in the development of degenerative diseases [10], atherosclerotic vascular disease [11], metabolic diseases [12], and various types of carcinomas [9]. At present, the research on circRNA in NSCLC is limited, and most of the research is to explore the circRNA that acts as a tumor driver [13], but the mechanism of circRNA for tumor suppression is still unclear. Therefore, exploring whether circRNAs can inhibit tumor progression is crucial for finding effective predictive biomarkers and actionable targets for intervention of NSCLC.

In this study, we analyzed the expression of circRNAs in NSCLC tissues by microarray and found that several circRNAs were abnormally expressed (mostly downregulated) in NSCLC. Through screening, we found that circ_0008602 was significantly downregulated in NSCLC tumor tissues and was significantly correlated with tumor size. Subsequent experiments demonstrated that circ_0008602, named circGUCY1A2, acts as a ceRNA to counteract miR-200c-3p-mediated PTEN inhibition, thereby inhibiting PI3K/AKT signaling pathway and NSCLC tumorigenesis. In addition, we found that the circadian gene ARNTL, which was reduced in NSCLC and could prolong the overall survival of patients, could bind to the promoter of circGUCY1A2, thereby increasing its expression. In conclusion, we identified a protective circGUCY1A2 that can be used as a potential diagnostic biomarker as well as a target for intervention in NSCLC.

## Methods and materials

### Patient and tissue samples

A total of 120 cancer tissues and paired adjacent non-tumor tissues were obtained from NSCLC patients during surgery at Xiangya Hospital of Central South University from May 2014 to December 2022. Patients did not receive any preoperative cancer treatments, such as radiotherapy or chemotherapy. Each specimen was rapidly frozen in liquid nitrogen and transferred to the − 80 °C refrigerator for subsequent experiments. The collected samples were confirmed by an experienced pathologist. The clinical data of NSCLC patients including tumor-node metastasis (TNM) staging were also collected (Table S1). Prior informed consents were obtained from the recruited patients and the study protocols were approved by Xiangya Hospital Medical Research Ethics Committee (No. 201,503,303).

### CircRNA extraction and detection

The purity and concentration of total RNA samples were determined with NanoDrop ND-1000. Then, total RNA from each sample was amplified and transcribed into fluorescent cRNA utilizing ramdom primer according to Arraystar Super RNA Labeling protocol (Arraystar, Inc.). Subsequently, the labeled cRNAs were hybridized onto the Arraystar Human circRNA Arrays (8 × 15 K, Arraystar), and incubated for 17 h at 65 °C in an Agilent Hybridization Oven. After washing, slides were scanned with the Agilent Scanner G25 05 C. The above processes are all completed by KangChen Bio-tech (Shanghai).

### Cell lines and cell culture

Two NSCLC cell lines (A549, PC-9 and H1299) and a normal human bronchial epithelial cell line (HBE) were purchased from the Chinese Academy of Sciences (Shanghai, China). Another NSCLC cell line H1703 was purchased from Cell Resource Center of COBIOER (Nanjing, China). PC-9 was cultured in RPMI Dulbecco’s Modified Eagle’s Medium (DMEM) supplemented with 10% fetal bovine serum (FBS) and 1% penicillin-streptomycin at 37 °C and 5% CO2. Other cell lines were cultured in RPMI1640 supplemented with 10% fetal bovine serum (FBS) and antibiotics. All cell lines were passaged less than 10 times after the initial revival from frozen stocks. All cell lines were authenticated prior to use by short tandem repeat profiling.

### RNA extraction and qRT-PCR analyses

Total RNA was isolated from frozen tumor specimens and cell lines using Trizol reagent (Invitrogen, Carlsbad, CA, USA) according to the manufacturer’s protocol. qRT-PCR assays were performed to detect targets expression using the Hifair III Super Mix cDNA synthesis and Hifair qPCR SYBR Green Master Mix (Cat No. 11,141 and 11,201; Yeasen, Shanghai, China). The relative levels of circGUCY1A2 and miR-200c-3p was determined by qRT-PCR using gene specific primers. The qRT-PCR reaction was performed in the ABI 7500 real-time PCR system (Applied Biosystems, CA, USA). The sequences of primers used in this study are shown in Table S2.

### Vector construction and transfection

The plasmids used in this study were constructed at Geneseed Biotech, Guangzhou, China. All vectors verified by sequencing. Virus packaging, production and cell transfection were performed according to the manufacture’s protocol. circGUCY1A2 interference vector, and the corresponding NC vector were obtained from RiboBio. The expression was validated by qRT-PCR.

### RNase R treatment

Split total RNA (20ug) to two aliquots, one for RNase R digestion with 3U/ug RNase R (Epicentre Technologies, Madison, WI, USA) and another for control with digestion buffer only. Incubate the samples at 37 °C for 30 min and purify the resulting RNA.

### CCK-8 assay

Cell proliferation was assessed by Cell Counting Kit-8 assay (Dojindo Laboratories, Kumamoto, Japan). The cells (1 × 10^3^) were seeded into 96-well plates. Then, 10 ml of CCK-8 solution was added to each well on days 1, 2, 3, 4 and 5. After 2 h of incubation at 37 °C, the absorbance at 450 nM was measured using an automatic microplate reader (Tecan, NANOQUANT, Swizerland). The experiment was repeated three times.

### Colony formation assay

For the colony formation assays, 1 × 10^3^ cells were plated in 6-well plates and incubated at 37 °C for 14 days. Colonies were dyed with dyeing solution containing 0.1% crystal violet and 20% methanol. Cell colonies were then counted and analyzed.

### 5-Ethynyl-20-deoxyuridine (EdU) incorporation assay

The EdU assay was performed with a Cell-Light EdU DNA Cell Proliferation Kit (RiboBio, Shanghai, China). Cells (1 × 10^4^) were seeded in each well of 96-well plates. After incubation with 50 mM EdU for 2 h, the cells were fixed in 4% paraformaldehyde and stained with Apollo Dye Solution. Hoechst 33,342 was used to stain the nucleic acid within the cells. Images were obtained with high-content imaging system (PERKINELMER, OPERETTA, USA), and the number of EdU-positive cells was counted.

### Luciferase reporter assay

3 × 10^4^ A549 cells were seeded in 24-well plates in triplicate [45]. 48 h after co-transfection with corresponding plasmids and microRNA mimics or inhibitors, luciferase reporter assays were conducted using Dual-luciferase reporter assay system (Promega, Madison, WI) according to the manufacturer’s instructions. Relative luciferase activity was normalized to the Renilla luciferase internal control.

### RNA immunoprecipitation

RNA immunoprecipitation (RIP) was performed by using the EZ-Magna RIP kit (Millipore, Billerica, MA). Approximately 1 × 10^7^ NSCLC cells were pelleted and re-suspended with an equal pellet volume of RIP Lysis Buffer (about 100 ml). The cell lysates (200 µl) were incubated with 5 µg of control rabbit IgG or antibody against Ago2 (Millipore). After treating with proteinase K buffer, the immunoprecipitated RNAs were extracted by RNeasy MinElute Cleanup Kit (Qiagen) and reversely transcripted using Prime-Script RT Master Mix (TaKaRa). The abundance of circGUCY1A2 and miR-200c-3p level was detected by qRT-PCR assay.

### Fluorescence in situ hybridization (FISH)

The hybridization was performed overnight with circGUCY1A2 and miR-200c-3p probes. Specimens were analyzed on a Nikon inverted fluorescence microscope. The circGUCY1A2 and hsa-miR-200c-3p probes for FISH are listed in Table S2.

### Western blotting

Cells were lysed in buffer containing 50 mM HEPES, 150 mM NaCl, 1 mM EDTA, 1% (w/v) CHAPS and Sigma protease inhibitor cocktail, and the total cell lysates were resolved with SDS-PAGE gels. The following antibodies were listed in Table S2.

### CRISPR/Cas9 system

CRISPR/Cas9 is mainly based on the LentiCRISPRv2 system, which uses BsmBI to digest the vector and can clone a pair of annealed oligos into a single-guide RNA scaffold. Oligos are designed based on the target site sequence (20 bp) and flanked at the 3’ end by PAM sequence. The sgPTEN sequence are listed in Table S2.

### Chip-qPCR

Chromatin immunoprecipitation assay (CHIP) was performed by using the Pierce Magnetic ChIP Kit (Thermo Scientific, America, USA). Chromatin was ultrasonically interrupted for 500 bp using M220 (covaries, America, USA). The primer sequences used in the CHIP assay are shown in Table S2.

#### Mouse models

Four-week-old BALB/C-nu nude male mice were used for animal studies, and all animals were maintained in the specific pathogen-free (SPF) conditions at our institution. For the in vivo tumor proliferation assay, 2 × 10^6^ A549 cells transfected with pLCDH-circGUCY1A2, or pLCDH-negative control were injected subcutaneously into the nude mice (5 per group). The tumor volume was monitored every week. After 6 weeks, the mice were sacrificed and tumor tissues were excised and subjected to pathologic and immunofluorescent examination.

Primary NSCLC samples were cut into approximately 1 mm^3^ fragments in 0.1 ml 50% Matrigel Basement Membrane Matrix (BD Biosciences) and directly implanted into the subcutaneous space (*n* = 5 for each tumor sample). Patient derived xenograft (PDX) tumors were harvested and divided into three portions for the generation of the second in vivo passage xenograft tumors. Mice with palpable tumors (approximately 200 mm^3^) were randomly divided into two experimental groups with intratumoral injection twice weekly for two weeks: 1.5 mg/kg pLCDH-circGUCY1A2 or pLCDH-negative control. At the end of the experiment, tumors were processed by qRT-PCR, pathologic examination and Immunohistochemistry. The results of immunohistochemistry were calculated by Image J. All mice were treated humanely and the Medical Experimental Animal Care Commission of Central South University approved all protocols for treating animals.

### Statistical analysis

Statistical analyses were performed using SPSS 17.0 (IBM, SPSS, Chicago, IL, USA) and GraphPad Prism. Paired t tests and Student’s t tests were used for paired and unpaired continuous data respectively. χ^2^ test was applied for categorical data. Survival curves were calculated using Kaplan-Meier method and Log-rank test, patients known to be alive at the time of the last follow-up were censored on the last date of contact. The differences between various groups were analyzed using Student’s t-test or assessed by one-way ANOVA. All the tests were two-sided and the value of *p* < 0.05 was considered to be statistically significant.

## Results

### circRNA expression profiles in NSCLC

**Fig. 1:**
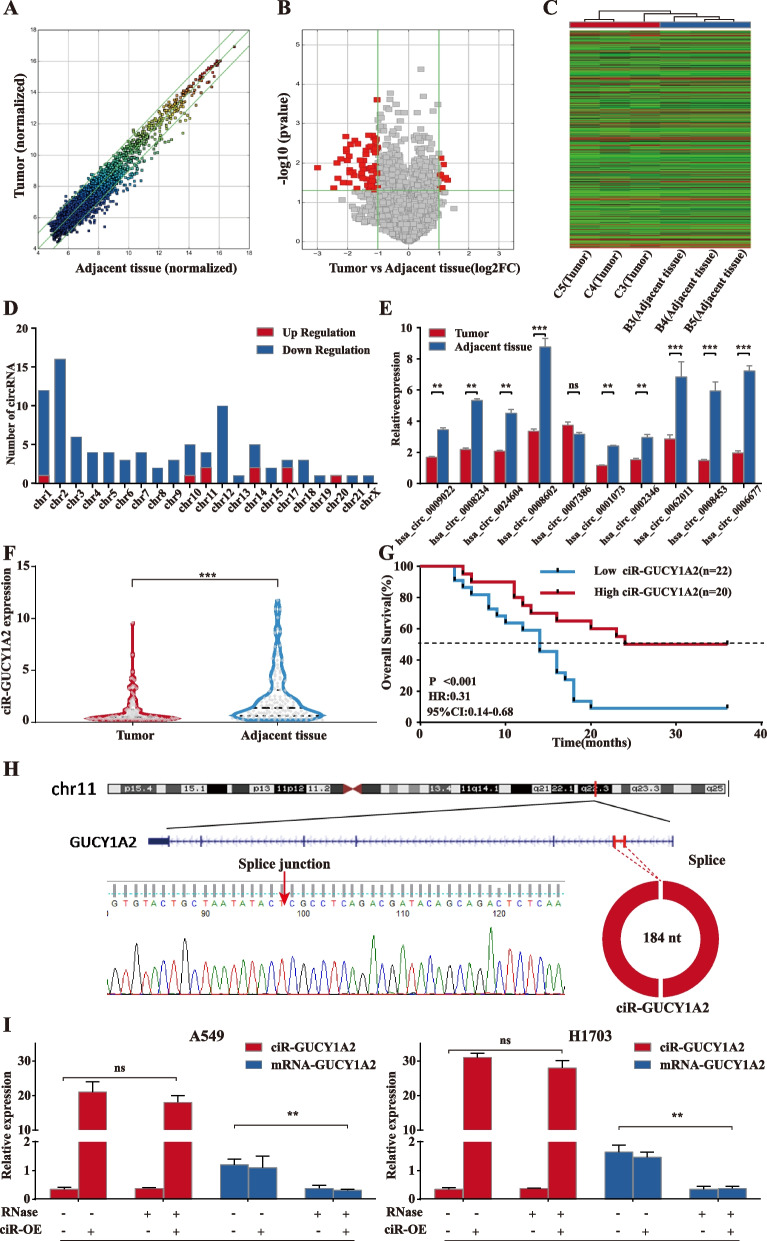
circRNA expression profile in NSCLC. **A** The scatter plot was used for assessing the variation in circRNA expression between tumor and paratumor samples. The values of x and y axes in the scatter plot were the normalized signal values of the samples (log2 scaled). **B** The volcano plot was constructed using fold-change values and *P*-values. The red dots represent differentially expressed circRNAs with statistically significant more than 2-fold changes. **C** The cluster heat map showed the differentially expressed circRNAs over 2-fold change. Red color indicates high expression level, and green color indicates low expression level. **D** Numbers of identified circRNAs in different chromosomes. **E** Expression of the top 10 downregulated circRNAs in tissues. **F** circGUCY1A2 expressions were evaluated using qRT-PCR in 120 pairs of NSCLC tissues and the matched adjacent normal tissues. **G** Kaplan-Meier survival curve indicated the high circGUCY1A2 expression is correlated with low survival rates. Data were represented as the mean ± SEM of three independent experiments. **H **The location of circRNA in chromatin, and its loop-forming site status **I** Total RNA extracted from the negative control or circGUCY1A2- transfected A549 and H1703 cells was incubated with or without RNAse R followed by real-time PCR. RNAse R treatment did not affect circGUCY1A2 levels but decreased linear GUCY1A2 mRNA levels. Ns: no significance, *P < 0.05, **P < 0.01, ***P < 0.001. Error bars indicate the S.E.M

First, we collected and analyzed circRNA expression signatures in 3 groups of NSCLC and adjacent tissues using microarrays [14]. The scatter and volcano plots showed the variation of circRNA expression between tumor and the adjacent tissues collected from the patients with NSCLC (Fig. 1A and B). The cluster heat map demonstrates the differentially expressed circRNAs over 2.0-fold change (Fig. 1C). The results showed that 92 circRNAs were significantly altered with fold change > 2.0 and *P* < 0.05, including 83 downregulated and 9 upregulated (Fig. 1D).

Subsequently, we performed quantitative reverse transcription polymerase chain reaction (qRT-PCR) on the top10 downregulated circRNAs to validate the microarray results. Consistent with the microarray results, as many as nine circRNAs were significantly downregulated in NSCLC tissues, with the highest degree of expression downregulation being circGUCY1A2 (Fig. 1E). By further expanding the tissue samples, a significant reduction in circGUCY1A2 expression was detected in 120 paired NSCLC tissues (Fig. 1F).

Analysis of clinical data showed that circGUCY1A2 expression was significantly correlated with tumor size and TNM stage (Table 1). And patients with higher circGUCY1A2 expression levels tended to have earlier TNM staging, as well as smaller tumors. Kaplan-Meier survival curves showed that reduced circGUCY1A2 expression was associated with poor survival in NSCLC patients (Fig. 1G). Therefore, we speculate that circGUCY1A2 may act as an important protective factor in lung adenocarcinoma and may be an intervention target and biomarker.

**Table 1:** circGUCY1A2 expression and clinicopathological features in non-small cell lung cancer (NSCLC) patients

Parameter	No. of patients	circGUCY1A2 (High)	circGUCY1A2 (Low)	*P* value
Gender				0.137
Male	69	21	48	
Female	16	8	8	
Age(year)				0.325
<60	53	16	37	
≥60	32	13	19	
Smoking history (years)				0.134
<10	29	13	16	
≥10	56	16	40	
Pathological type				0.090
adenocarcinoma	39	17	22	
squamous carcinoma	46	12	34	
Tumor size (cm)				< 0.001***
≤5	45	23	22	
>5	40	6	34	
Differentiation Grade				0.455
Well-moderate	51	19	32	
Poor-undifferentiation	34	10	24	
TNM stage				0.043*
I-II	55	23	32	
III-IV	30	6	24	
Metastasis				0.5228
Yes	22	5	13	
No	63	24	43	

Footnote: The TNM Staging System is based on the tumor (T), the extent of spread to the lymph nodes (N), and the presence of metastasis (M)*P < 0.05, ***P < 0.001

According to CircBase database annotation, circGUCY1A2 is spliced from GUCY1A3 gene on chr11: 106,849,344–106,856,857 and ultimately formed the length of 184 nt (Fig. 1H). Then, the overexpression vector of circGUCY1A2 was constructed to further investigate its biological function. By transfecting the vector and performing RNase digestion experiments, we demonstrated that the vector can significantly increase the expression of circGUCY1A2 without increasing the expression of linear RNA. Meanwhile, the circGUCY1A2 has a closed loop structure and is more stable compared to linear RNA (Fig. 1I).

### circGUCY1A2 play a tumor suppressive role in NSCLC cells

**Fig. 2:**
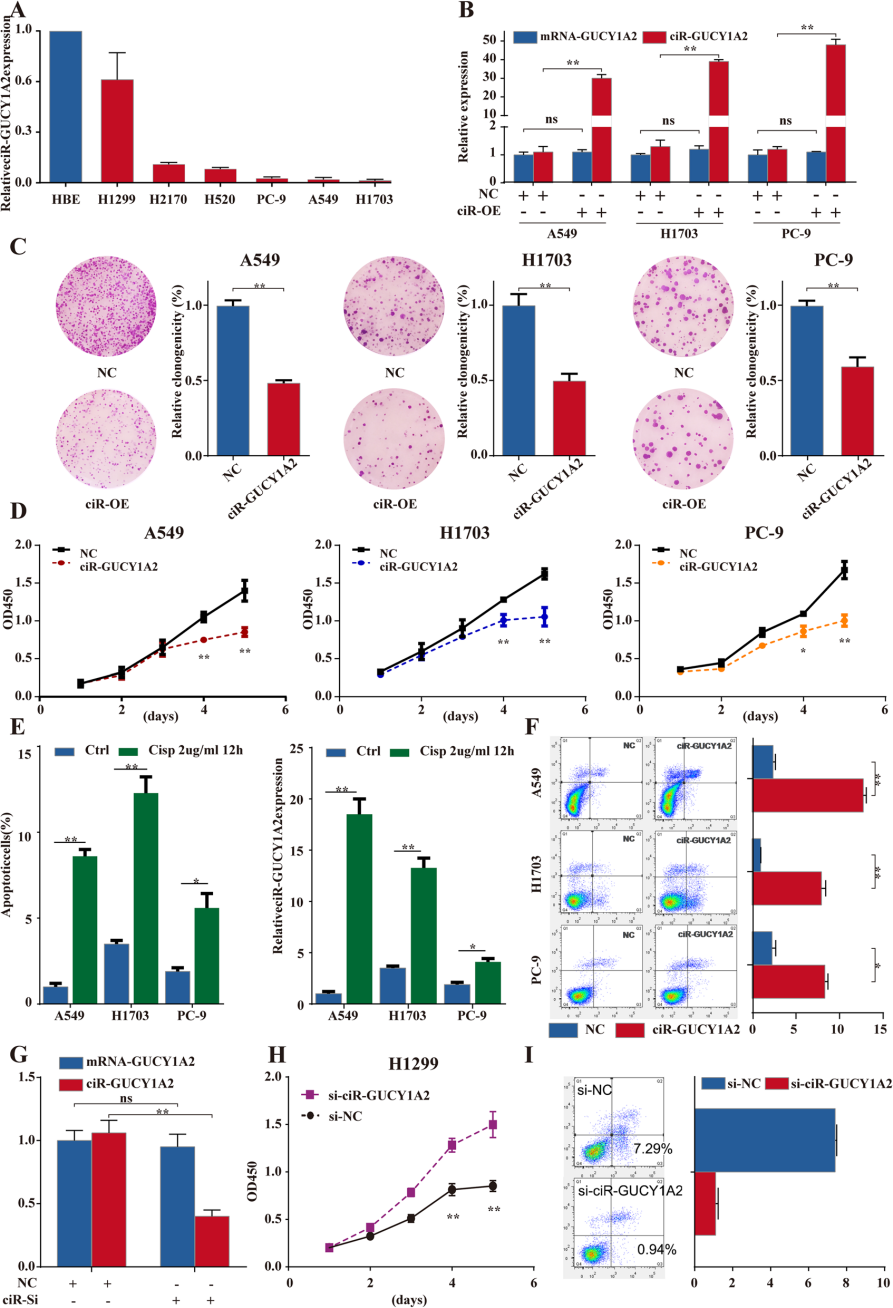
circGUCY1A2 exerts suppressive effects in NSCLC cells. **A** circGUCY1A2 expression in NSCLC cell lines and normal human bronchial epithelial cell lines. **B** The expressions of mRNA-GUCY1A2 and circGUCY1A2 were determined with qPCR in NSCLC cells transfected with negative control or pLCDH-circGUCY1A2. **C** Colony formation assay of A549, H1703 and PC-9 cells transfected with negative control or pLCDH-circGUCY1A2. **D** Assessment of proliferation of A549, H1703 and PC-9 cells transfected with negative cotnrol or pLCDH-circGUCY1A2 by CCK-8 assay. **E** NSCLC cell lines A549, H1703,and PC-9 were cultured in the presence of Cisplatin. The cells were subject to Annexin-V staining to detect apoptosis. Treatment with Cisplatin increased apoptosis. RNAs were isolated and subject to real-time PCR to measure circGUCY1A2 levels. **F** Apoptosis rate was analyzed by flow cytometry. NSCLC cells were transfected with negative control or pLCDH-circGUCY1A2. circGUCY1A2 enhanced cell apoptosis in NSCLC cells. **G** The expressions of mRNA-GUCY1A2 and circGUCY1A2 were determined with qPCR in H1299 cells transfected with negative control or si-circGUCY1A2. **H** Assessment of proliferation of H1299 cells transfected with negative cotnrol or si-circGUCY1A2 by CCK-8 assay. **I** Apoptosis rate was analyzed by flow cytometry. H1299 cells were transfected with negative control or si-circGUCY1A2. NC: negative control, scrambled sequence. Data were represented as the mean ± SEM of three independent experiments. Ns: no significance, *P < 0.05, **P < 0.01, ***P < 0.001. Error bars indicate the S.E.M

To select cell lines for subsequent experiments, we examined circGUCY1A2 expression in six NSCLC cell lines (H1299, H2170, H520, PC-9, A549 and H1703). The results showed that circGUCY1A2 were downregulated in NSCLC cell lines compared to the normal human bronchial epithelial cell line HBE (Fig. 2A). Therefore, we transfected pLCDH-circGUCY1A2 or scrambled sequence (negative control) into A549, H1703 and PC-9 NSCLC cells, which had lower basal circGUCY1A2 expression levels. The qRT-PCR analysis indicated that the vector transfection significantly increased circGUCY1A2 expression without affecting linear expression of GUCY1A2 (Fig. 2B). We first examined the effect of circGUCY1A2 on cell proliferation, and colony formation assays showed that the proliferation of A549, H1703 and PC-9 cells were all significantly inhibited by the overexpression of circGUCY1A2 (Fig. 2C). meanwhile, CCK-8 assay showed that overexpression of circGUCY1A2 impaired the proliferation of NSCLC cells (Fig. 2D). In addition, we also constructed plasmids to explore the biological functions of the parental GUCY1A2 gene. CCK-8 assay showed that overexpression of GUCY1A2 did not significantly affect the proliferation of NSCLC cells (Fig. S1).

Subsequently, we exposed three NSCLC cell lines to 2 ug/ml cisplatin for 12 h and subsequently examined the apoptosis of the cells and the expression of circGUCY1A2. The results showed that the expression of circGUCY1A2 was significantly increased in cells exposed to cisplatin, which was consistent with an increase in cell death (Fig. 2E). Next, we explored whether circGUCY1A2 enhanced apoptosis in NSCLC cells. The above three NSCLC cell lines were transfected with pLCDH-circGUCY1A2 or negative control, and the apoptosis of NSCLC cells was measured by flow cytometry. The results showed that overexpression of circGUCY1A2 enhanced apoptosis in A549, H1703 and PC-9 cells (Fig. 2F).

In addition, we also verified whether knockdown of circRNA influences cell proliferation and apoptosis. We transfected the specific small interfering RNA (siRNA) against circGUCY1A2 into H1299 cells, which had high levels of basal circGUCY1A2 expression, and the results showed that siRNA significantly reduced circGUCY1A2 expression without affecting linear RNA expression (Fig. 2G). As expected, CCK-8 assay showed that knockdown of circGUCY1A2 promoted the proliferation of H1299 compared to the negative control group (Fig. 2H). The apoptosis of H1299 was also measured by flow cytometry, and the results showed that knockdown of circGUCY1A2 suppressed the apoptosis of H1299 (Fig. 2I). Overall, the above results suggest that circGUCY1A2 may play a tumor suppressor role in NSCLC by inhibiting cell proliferation and increasing apoptosis.

### circGUCY1A2 inhibits NSCLC progression by directly binding to miR-200c-3p

**Fig. 3:**
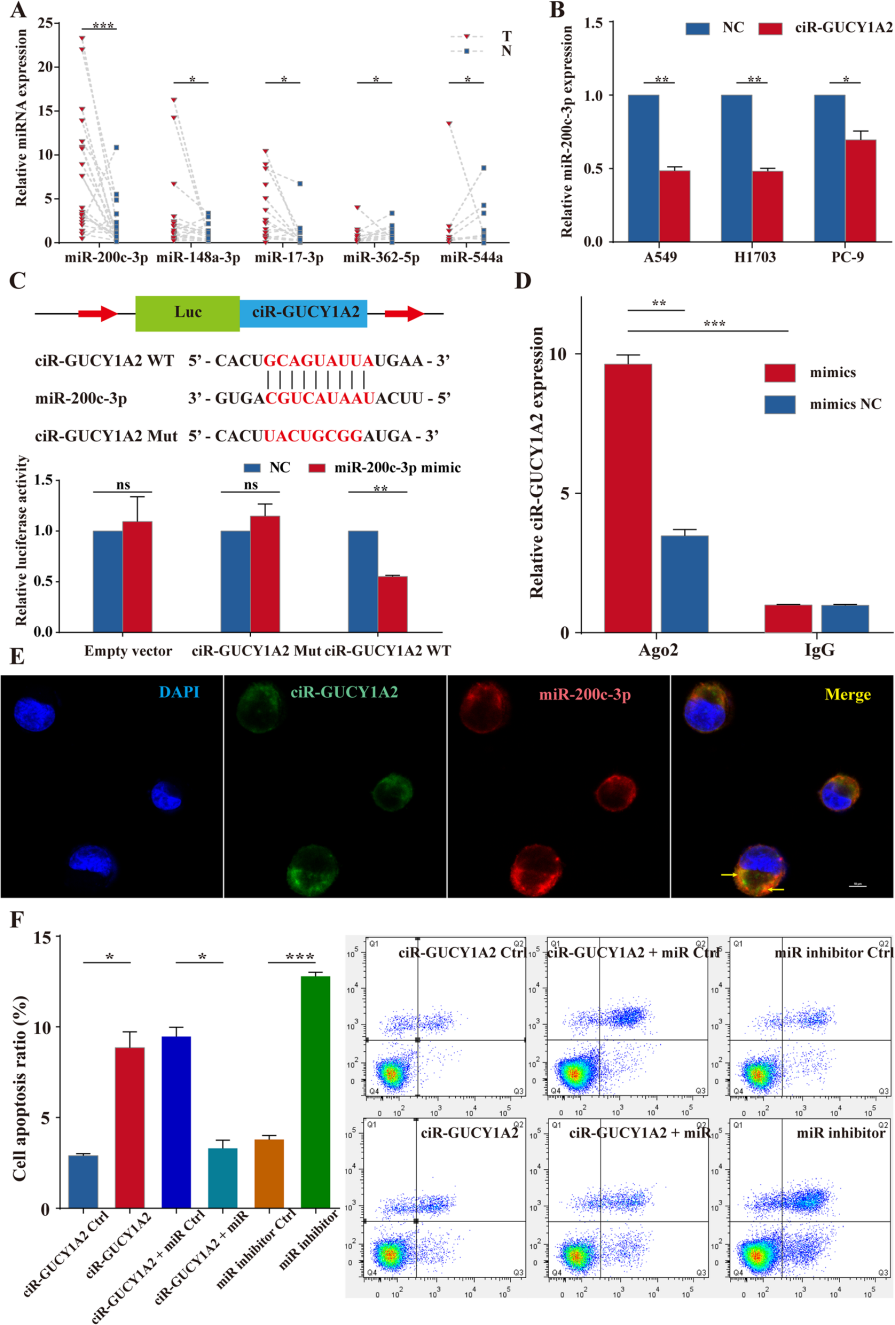
circGUCY1A2 affects NSCLC apoptosis by binding miR-200c-3p **A** Relative expression of the five indicated miRNAs from 20 NSCLC tumor tissues and the matched adjacent normal tissues. T: tumor; N: nontumor. **B** The expressions of miR-200c-3p were analyzed using qRT-PCR in NSCLC cells transfected with negative control or pLCDH-circGUCY1A2. **C** Schematic of circGUCY1A2 wild-type (WT) and mutant (Mut) luciferase reporter vectors. The relative luciferase activities were analyzed in A549 cells co-transfected with miR-200c-3p mimics or miR-NC and luciferase reporter vectors pLCDH-circGUCY1A2- WT or pLCDH-circGUCY1A2- Mut. **D** AGO2 RNA immunoprecipitation (RIP) assay for circGUCY1A2 and miR-200c-3p levels in NSCLC cells. **E** miR-200c-3p co-localized with cirR-GUCY1A2 in H1299 was detected by FISH, Magnification, 400×. **F **After overexpression of circGUCY1A2, miR-200c-3p mimic was added to A549 and H1703 cell culture. Apoptosis ratio of NSCLC cells was measured by fluorescent-activated cell sorting. Data were represented as the mean ± SEM of three independent experiments. Ns: no significance, *P < 0.05, **P < 0.01, ***P < 0.001. Error bars indicate the S.E.M

To explore the mechanism by which circGUCY1A2 function as tumor suppressors, we searched for candidate target genes of circGUCY1A2 using public databases, including StarBase (v2.0). Subsequently, the five candidates (miR-148a-3p, miR-362-5p. miR-200c-3p, miR-544a, miR-17-3p) with the highest predictive scores were selected for detection in tissues from matched lung adenocarcinoma patients. QRT-PCR results showed that miR-200c-3p was most upregulated in NSCLC tissues than in adjacent normal tissues (Fig. 3A).

Based on the mechanism of circRNA as RNA sponge, we selected 3 up-regulated miRNAs as research objects. After overexpressing circGUCY1A2, we found that miR-200c-3p was most significantly upregulated, followed by miR-148a-3p, while miR-17-3p expression did not change significantly (Fig. 3B and Fig. S2A). Further, a dual-luciferase reporter system was used to confirm whether miR-200c-3p and miR-148a-3p were direct targets of circGUCY1A2. Plasmids containing mutant sequence and wild-type sequences were designed according to the binding sites and were co-transfected with miR-200c-3p and miR-148a-3p in 293T cells. The results showed that there was no significant variation in luciferase activity was observed for either the circGUCY1A2-Mut or the negative control co-transfected with miR-200c-3p-mimic, while the reporter activity of circGUCY1A2-WT co-transfected with miR-200c-3p-mimic was significantly decreased (Fig. 3C). At the same time, although miR-148a-3p was predicted to have a binding site with circGUCY1A2, no significant change in reporter activity was observed among the three groups, which may be due to weak binding ability (Fig. S2B). Thus, the luciferase assays revealed that miR-200c-3p could bind to the circGUCY1A2, causing a significant decrease in luciferase activity compared with the negative control. Subsequently, RIP assays were performed to investigate whether circGUCY1A2 and miR-200c-3p were involved in the expected RNA-induced silencing complex (RISC). The results showed that anti-Ago2 group could greatly pull down the circGUCY1A2 compared to the anti-IgG group. In addition, the expression of circGUCY1A2 was superior in cells transfected with miR-200c-3p mimics than in cells transfected with miR-200c-3p mimics NC (Fig. 3D). Then, the subcellular localization of circGUCY1A2 and miR-200c-3p was observed by the FISH assay in H1299 cells. As shown in Fig. 3E, most of the circGUCY1A2 (green) and miR-200c-3p (red) were placed in the cytoplasm. The arrow in the merge figure suggested that circGUCY1A2 and miR-200c-3p may bind in the cytoplasm.

**Fig. 4:**
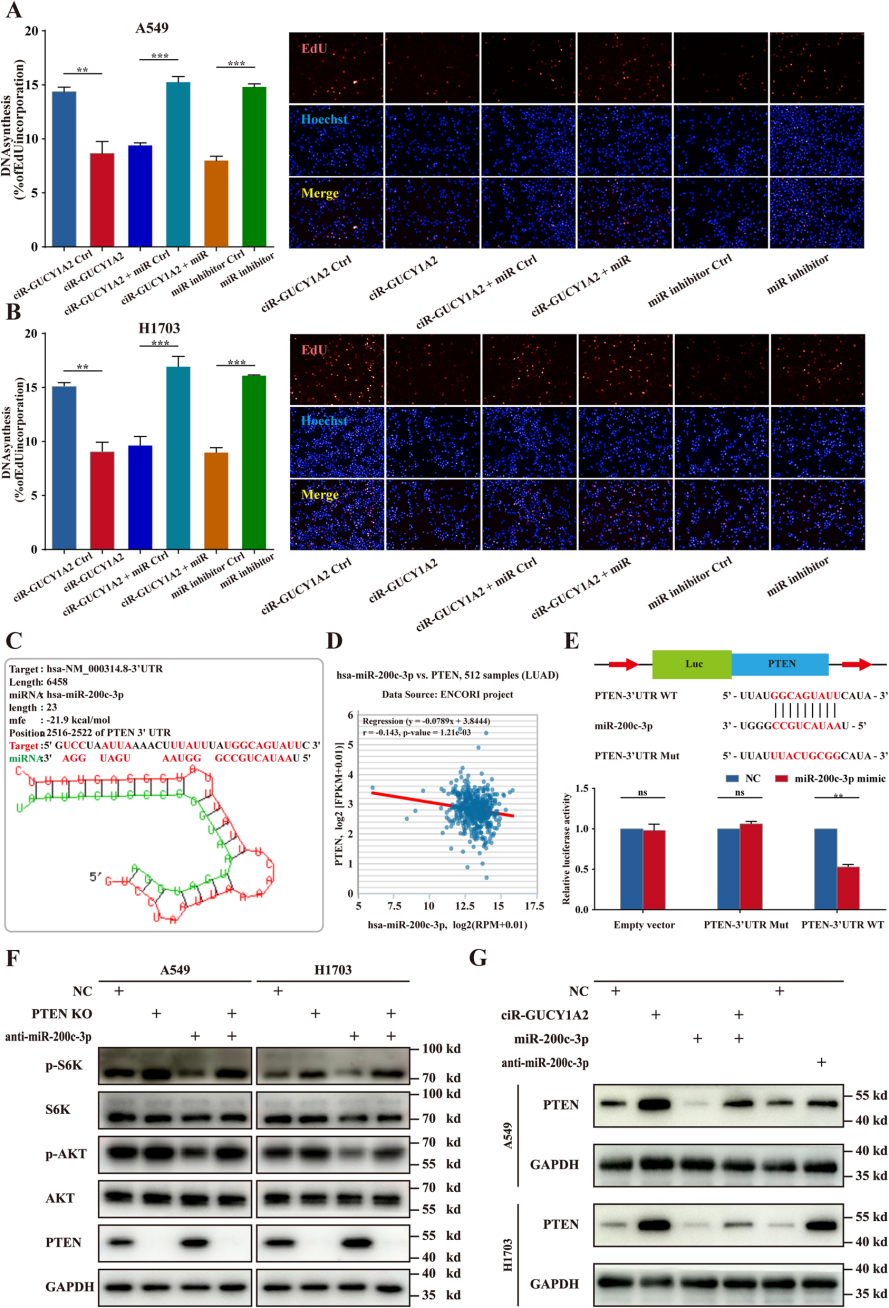
miR-200c-3p affects lung adenocarcinoma through regulation of PTEN. **A**-**B** After overexpression of circGUCY1A2, miR-200c-3p mimic was added to A549 and H1703 cell culture. Proliferation ratio of NSCLC cells was measured by EdU incorporation. **C** Location and structural information of miR-200c-3p. **D** Correlation analysis between miR-200c-3p and PTEN expression in patients with lung adenocarcinoma. **E** Schematic of the construction of wild-type or mutant pGL3-PTEN 3’-UTR vectors is illustrated. Relative luciferase activity was analyzed in A549 cells. Firefly luciferase vector was used as an internal control. **F** Inhibiting miR-200c-3p and knocking out PTEN to observe the protein level of PTEN/PI3K/AKT. **G** After overexpression of circGUCY1A2, miR-200c-3p mimic was added to A549 and H1703 cell culture. The PTEN protein expression level was analyzed by Western blot. Data were represented as the mean ± SEM of three independent experiments. Ns: no significance, *P < 0.05, **P < 0.01, ***P < 0.001. Error bars indicate the S.E.M

Upregulation of miR-200c-3p has been found in various types of cancer, and high expression of miR-200c-3p is associated with a more aggressive phenotype [15–17]. To investigate whether circGUCY1A2 exerts its anti-tumor effect through the sponge activity of miR-200c-3p, we detected the apoptosis and proliferation of NSCLC cells co-transfected with circGUCY1A2 and miR-200c-3p. The results of flow cytometry demonstrated that miR-200c-3p could reverse the effect of circGUCY1A2 on promoting apoptosis. At the same time, the use of miR-200c-3p inhibitors also significantly increased cell apoptosis (Fig. 3F). In EdU analysis, circGUCY1A2 overexpression decreased the percentage of EdU-positive cells, and this trend was reversed after miR-200c-3p co-transfection. Besides, the use of miR-200c-3p inhibitors also significantly increased the percentage of EdU-positive cells (Fig. 4A and B). These findings indicate that circGUCY1A2 indeed play a role in inhibiting proliferation and promoting apoptosis in NSCLC cells partly through miR-200c-3p.

### PTEN is a direct target of miR-200c-3p in NSCLC

Three bioinformatics databases (TargetScan, miRanda, and RNAhybrid) were used to predict the target genes that miR-200c-3p could bind (Table S3). According to our previous research and literature review, we screened several tumor suppressor genes (PTEN, NOTCH1, p73 and KLF4, etc.) for pre-experimental screening. By overexpressing and knocking down miR-200c-3p and examining the expression of these potential targets, we found that the expression of PTEN changed most significantly, followed by NOTCH1, while the expression of p73 and KLF4 had no significant changes (Fig. S3). Therefore, PTEN was identified as the direct target, and the binding site was at 2516–2522 of PTEN 3’UTR (Fig. 4C). Besides, data from the ENCORI project containing 512 samples showed that miR-200c-3p was significantly negatively correlated with the expression of PTEN, suggesting that miR-200c-3p could reduce the expression of PTEN (Fig. 4D).

Subsequently, we designed plasmids containing PTEN 3’UTR mutant sequences and wild-type sequences based on the dual-luciferase reporter system, and co-transfected them with miR-200c-3p in 293T cells. We found that that overexpression of miR-200c-3p resulted in decreased luciferase activity of PTEN 3’-UTR-WT, while luciferase activity of PTEN 3’-UTR-MUT had no significant change (Fig. 4E). This result directly proved that miR-200c-3p could directly bind to PTEN 3’-UTR.

Furthermore, PTEN knockout (KO) cell line was constructed using CRISPR/Cas9 system to explore the biological process of miR-200c-3p/PTEN. The sequencing data of previous researchers in the Cancer Cell Line Encyclopedia (CCLE) database showed that A549 and H1703 had no PTEN mutation and copy number variation and were therefore selected for follow-up studies. CCK8 rescue experiments showed that inhibition of miR-200c-3p could significantly inhibit the proliferation of tumor cells, and this trend was reversed when PTEN was knockout (Fig. S4A-B). Therefore, miR-200c-3p does exert its biological function partially through PTEN. The western blot (WB) results showed that inhibition of miR-200c-3p could increase PTEN protein expression, thereby inhibiting the activation of S6K phosphorylation (Thr389) and AKT phosphorylation (Ser473). This pathway is rescued when PTEN is knocked out (Fig. 4F). In addition, we also explored the effects of circGUCY1A2 and miR-200c-3p on PTEN at the protein level. WB results showed that circGUCY1A2 could increase the protein level of PTEN, while miR-200c-3p could decrease the protein level of PTEN. Importantly, co-transfection of miR-200c-3p could reverse the effect of circGUCY1A2 on PTEN protein level (Fig. 4G). The grey value analysis is shown in Figs. S4C and D.

Moreover, we also detected the expression of PTEN in our own clinical tissues. The results showed that the expression of PTEN was significantly upregulated in patients with high expression of circGUCY1A2 (Fig. S4E). However, high expression of PTEN had no significant effect on the prolongation of overall survival of patients. (Fig. S4F). We will continue to increase the data scale in future work to explore whether PTEN can independently affect the prognosis of patients. Based on the above results, we reasonably speculate that circGUCY1A2 acts as a miRNA sponge and competitively binds with miR-200c-3p, thereby eliminating the inhibitory effect of miR-200c-3p on PTEN and ultimately inhibiting the progression of NSCLC through PI3K/AKT signaling pathway.

### Overexpression of circGUCY1A2 suppresses NSCLC growth in vivo

**Fig. 5:**
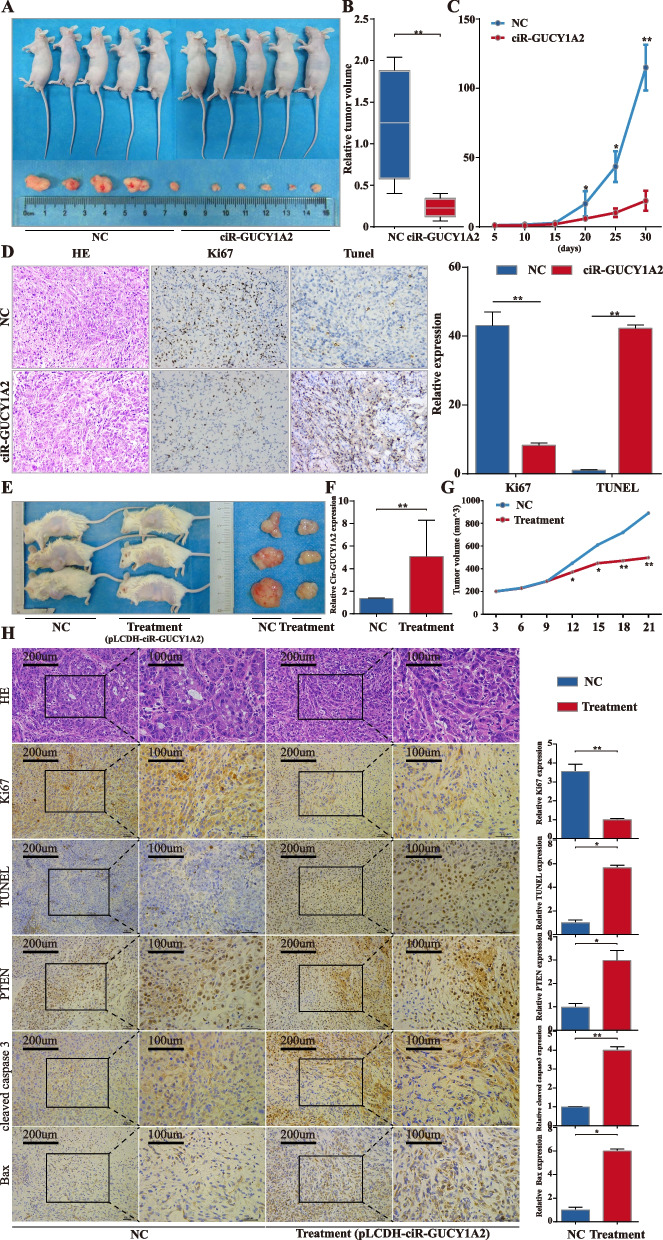
circGUCY1A2 is a potential therapeutic target. **A** Representative images of the tumor bearing BALB/c nude mice and xenograft tumors. **B** The relative volumes of the tumors in the circGUCY1A2 groups and control groups. **C** Tumor growth curves of subcutaneous implantation models of NSCLC. **D** Representative images of HE and immunohistochemical staining for TUNEL and Ki67 expression in tumor tissues (original magnification: 200×). **E** Representative images of tumor bearing nod/scid mice PDX model tumors. **F** circGUCY1A2 expression of PDX model tumors. **G** Growth curves of PDX model tumors. **H** Immunohistochemistry staining of PTEN, Ki67, TUNEL, cleaved-caspase 3 and Bax. NC: negative control (original magnification: 200× and 400×). Ns: no significance, *P < 0.05, **P < 0.01, ***P < 0.001. Error bars indicate the S.E.M

The results of in vitro experiments preliminarily prove that circGUCY1A2 inhibits proliferation and promotes apoptosis of NSCLC cells through the miR-200c-3p/PTEN axis, but the tumorigenic effect of circGUCY1A2 in vivo is still unclear. Lentivirus-oe-circGUCY1A2 was transfected into A549 cells to construct a stable expression cell line. Subsequently, cells stably expressing circGUCY1A2 and negative vector were injected subcutaneously into nude mice. Palpable tumors formed within 6 weeks after implantation. Tumor volume was measured every 5 days, and mice were sacrificed 30days after tumor cell implantation (Fig. 5A). The size of NSCLC tumors in these two groups was calculated and compared. The average tumor volume of A549 cells stably transfected with circGUCY1A2 was 0.21 ± 0.077 cm^3^, which was significantly smaller than tumors in the negative control group (1.22 ± 0.705 cm^3^) (Fig. 5B). The tumor growth-curve of tumor volume was drawn according to time and a significant difference was shown between the two groups (Fig. 5C). Subcutaneous tumor tissues were collected, and Immunohistochemistry (IHC) assays showed that upregulation of circGUCY1A2 increased TUNEL expression and decreased Ki67 expression in xenograft tumor tissues (Fig. 5D).

In addition, we then developed a PDX model from lung adenocarcinoma patients and evaluated the therapeutic potential of circGUCY1A2 by intratumoral injection of pLCDH-circGUCY1A2 or pLCDH-negative control (4 times and twice a week) (Fig. 5E). PDX tumor tissues were collected, and RT-PCR results showed that the expression of circGUCY1A2 in the treatment group was significantly higher than that in the NC group (Fig. 5F). At the same time, the tumor growth in the treatment group was significantly inhibited (Fig. 5G). IHC assays revealed that tumor tissues collected from the circGUCY1A2 group had more PTEN, TUNEL, cleaved-caspase3 and Bax positive cells but fewer Ki67 positive cells when compared with control group (Fig. 5H). Taken together, circGUCY1A2 can inhibit tumor growth in vivo and has the potential to be used as a clinical therapeutic target.

### ARNTL is responsible for the low expression of circGUCY1A2

**Fig. 6:**
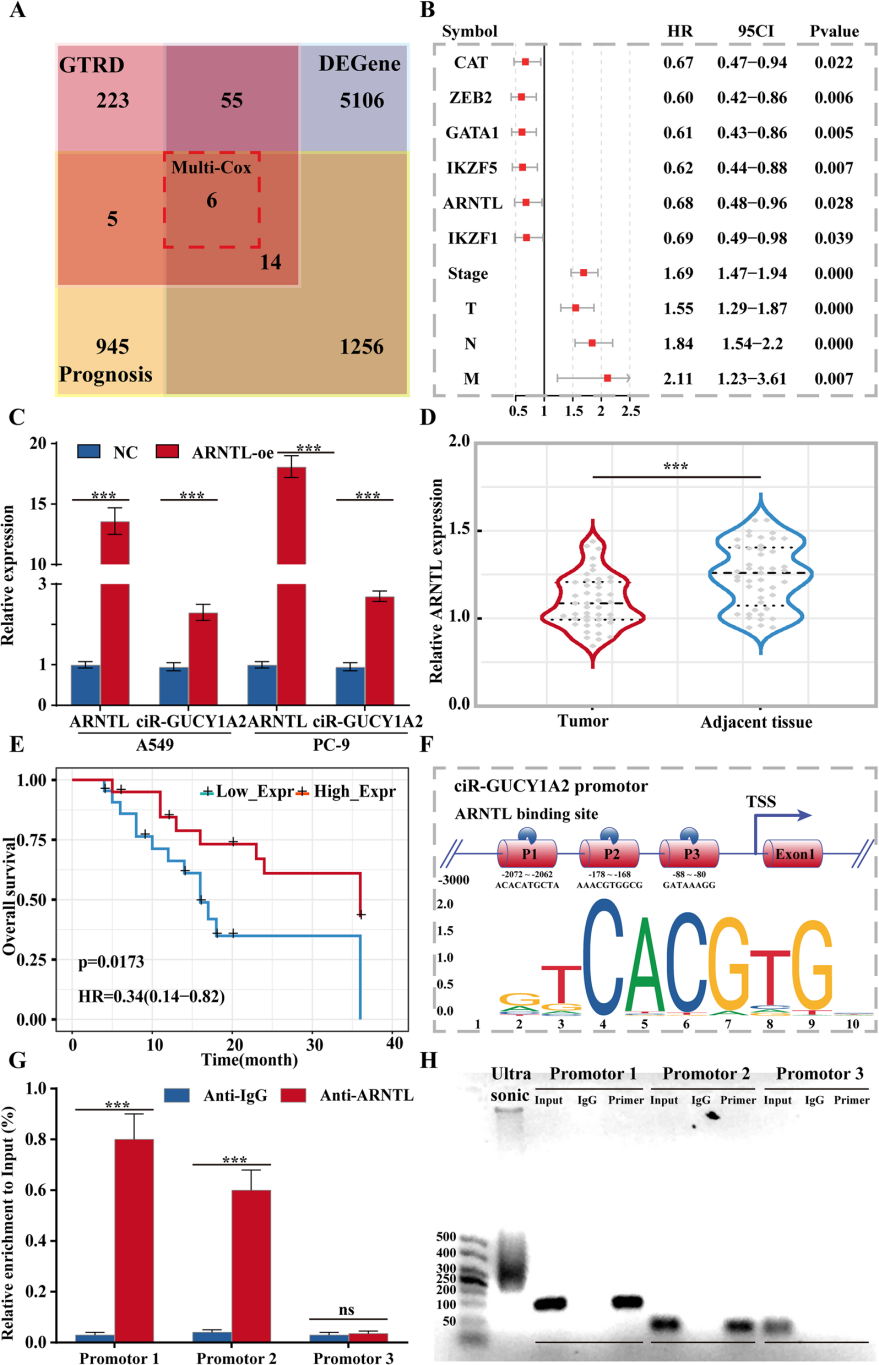
ARNTL can initiate transcription of circGUCY1A2. **A** Identification of tumor-specific and prognostically relevant transcription factors through GTRD and TCGA databases. **B** Forest plots of core tumor-specific and prognostically relevant transcription factors and clinical indicators. **C** ARNTL overexpression in A549 and PC-9 can significantly increase circGUCY1A2 expression. **D** ARNTL expressions were evaluated using qRT-PCR in NSCLC tissues and the matched adjacent normal tissues. **E** Kaplan-Meier survival curve indicated the low ARNTL expression is correlated with low survival rates. Data were represented as the mean ± SEM of three independent experiments. **F** Prediction of ARNTL three binding sites and motif to GUCY1A2. **G** CHIP-qPCR results suggest that ARNTL may bind to promoter and promoter 2. **H** Gel electrophoresis was performed to demonstrate information of DNA sonication interruption and qRT-PCR products. Ns: no significance, *P < 0.05, **P < 0.01, ***P < 0.001. Error bars indicate the S.E.M

We have comprehensively demonstrated that low expression of circGUCY1A2 causes abnormal tumor proliferation by the in vivo and in vitro experiments, but it remains unclear who is responsible for circGUCY1A2 low expression. GTRD is a public database that summarizes the results of chip-seq experiments from published studies. A total of 298 transcription factors (TFs) were found to potentially regulate circGUCY1A2 expression by reviewing the GTRD database. By analyzing differentially expressed genes and prognosis-related genes in TCGA lung adenocarcinoma patients, we screened for 20 transcription factors that may be involved in the regulation of circGUCY1A2 (Fig. 6A). Subsequently, we combined each of these 20 genes separately with prognosis-related clinical features for multivariate regression analysis to find independent prognostic signature of clinical value. Ultimately, six protective factors were screened as able candidates for our follow-up study (Fig. 6B).

Subsequently, plasmids containing these genes were constructed in batches and transfected into tumor cells. Interestingly, overexpression of ARNTL was found to significantly increase the expression of circGUCY1A2 by qRT-PCR (Fig. 6C and Fig. S5). In addition, we found that ARNTL was significantly downregulated in lung adenocarcinoma tissues by examining our own clinical samples (Fig. 6D), and that patients with low ARNTL expression had a poorer prognosis (Fig. 6E).

To elucidate the specific mechanism by which ARNTL regulates circGUCY1A2, we have predicted promoter binding sites through multiple databases (Ensemble, GTRD and JASPAR), and selected three of them with high confidence scores for further study (Fig. 6F). Relevant specific primers were designed to target these binding sites, and chip-qPCR results showed that ARNTL could bind to promoter 1/2, with a stronger signal from promoter 1 (Fig. 6G). Gel electrophoresis was performed to demonstrate information of DNA sonication interruption and qRT-PCR products (Fig. 6H). The results suggested that ARNTL could increase the expression of circGUCY1A2 by binding to its chromatin promoters.

## Discussion

CircRNA, a non-coding RNA with a stable structure, was thought to be rare and reflect errors in the last century [18]. It is only in recent years that its importance has gained attention, especially in the field of cancer research, and has become a new and unique class of epigenetic regulators with roles in cancer [19]. Current research on circRNA has mostly demonstrated its ability to maintain cell stemness, differentiation, invasion, migration, angiogenesis, and other aspects that promote tumor development [20]. However, few studies have investigated circRNA as a protective factor against tumor progression and as a possible clinical intervention agent.

In this study, we first found that circGUCY1A2 was significantly downregulated in lung tumor tissues and its expression was associated inversely to tumor size of NSCLC. Through in vivo and in vitro experiments, we systematically demonstrated that overexpression of circGUCY1A2 inhibited NSCLC cell proliferation and promoted apoptosis of NSCLC cells. Further in-depth mechanistic studies revealed that circGUCY1A2 can act as ceRNA to compete for binding miR-200c-3p and exert tumor suppressive effects.

MiR-200c is a member of miR-200 family with 4 other family members (miR-200a, miR-200b, miR-429 and miR-141) located in chromosome 12 (12q13.31) together with miR-141. miR-200c-3p is the mature miRNA of miR-200c [15]. Upregulation of miR-200c-3p was found in various types of cancers and high miR-200c-3p expression was associated with more aggressive phenotypes [21–24], but studies on miR-200c-3p in lung adenocarcinoma are scarce. MiR-200c-3p was predicted to interact with circGUCY1A2 by bioinformatics and confirmed by fluorescent reporter system and fluorescent in situ hybridization. Follow-up EdU and flow cytometry assays demonstrate that miR-200c-3p can reverse the biological function of circGUCY1A2.

PTEN, as a tumor suppressor factor, was found to be dysregulated at a high frequency in many cancers [25–34]. Data from the TCGA database showed that the frequency of PTEN mutations and deletions was only 2.4% in lung adenocarcinoma and 21% in lung squamous cell carcinoma (Fig. S6). Therefore, the reason for the downregulation of PTEN in most patients is not clear, and our study may serve as an important supplement for PTEN inactivation in NSCLC. PTEN exerts tumor suppression mainly through its lipid phosphatase activity, which opposes the activation of PI3K/AKT [35]. Previous studies have found that the downregulation of PTEN is mostly attributed to high mutation frequency [25–28], and it remains to be investigated whether there are other ways to regulate. Our study identified and confirmed that miR-200c-3p was able to reduce PTEN expression by fluorescent reporter system and WB, while circGUCY1A2 overexpression could reverse this phenomenon.

**Fig. 7:**
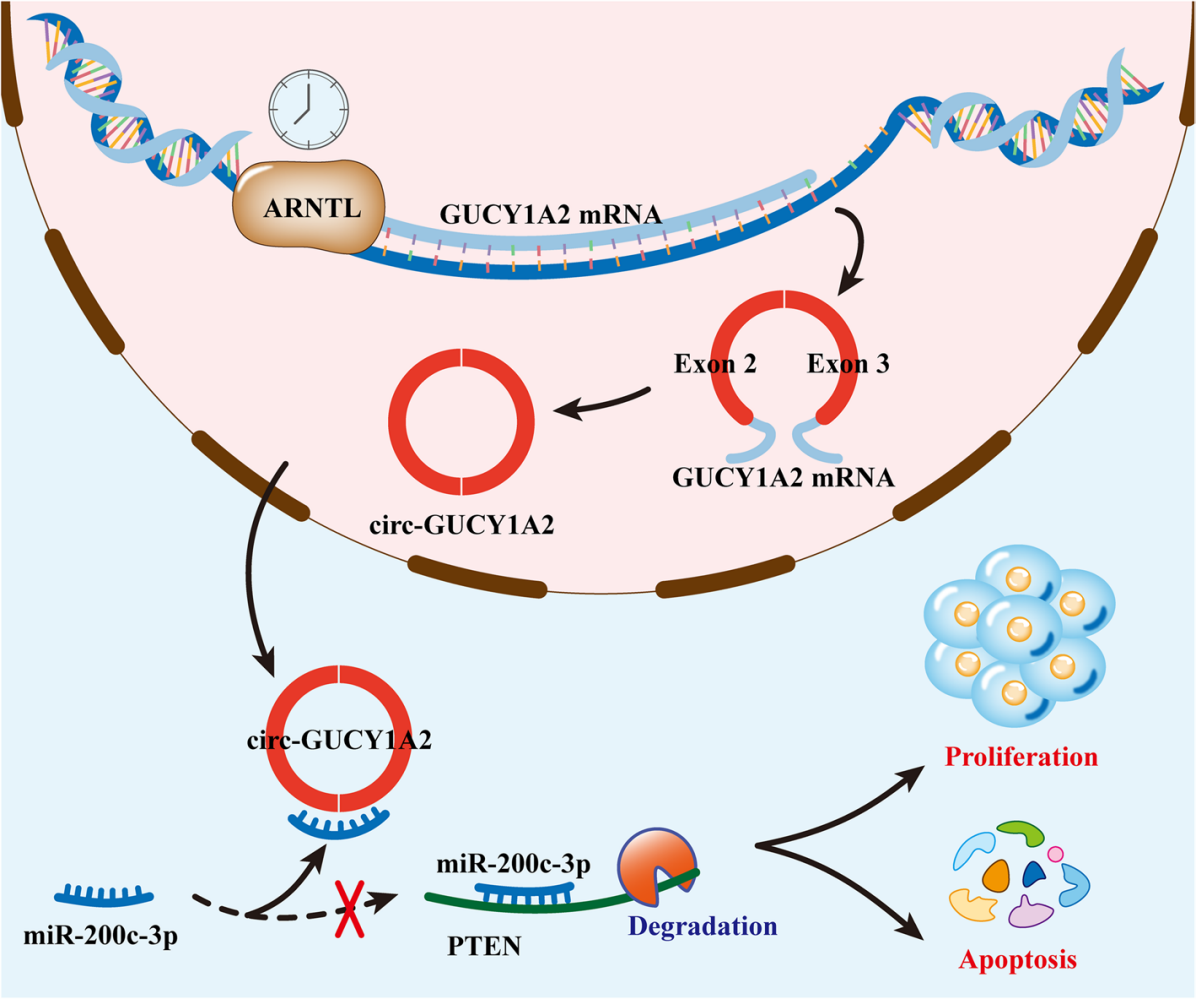
The schematic diagram shows the mechanism underlying ARNTL can inhibit the development of lung adenocarcinoma through the circGUCY1A2/miR-200c-3p/PTEN axis

Time is a core element of all biological process. All organisms have conserved time systems designed to assist circadian mechanisms and molecular pathways that control cell division [36]. ARNTL, one of the core mammalian clock molecules, serves as a key transcription factor that regulates ~ 10% of cellular gene expression [37]. Thales’ study showed that the central circadian gene ARNTL has cell-autonomous tumor suppressive effects in the progression and transformation of lung tumors [38]. The specific mechanism by which ARNTL inhibits lung adenocarcinoma progression remains to be refined. Our study elucidates for the first time that ARNTL may inhibit tumor development through a non-coding RNA pathway. In detail, ARNTL increases the expression of circGUCY1A2 by binding to the promoter of GUCY1A2. Subsequently, circGUCY1A2 promotes the expression of PTEN molecules by acting as a sponge-binding miR-200c-3p, which ultimately exerts tumor suppressive effects (Fig. 7). This discovery, which fills in the molecular mechanism of the role of ARNTL in lung adenocarcinoma, may provide potential targets and therapeutic options for the treatment of lung adenocarcinoma. However, why ARNTL is dysregulated in lung adenocarcinoma requires our further study.

## Conclusions

In summary, we demonstrate that the circadian gene ARNTL initiates circGUCY1A2 transcription and suppresses lung adenocarcinoma progression through the miR-200c-3p/PTEN axis. Our research helps to clarify the potential inhibitory pathways in lung adenocarcinoma and provide biological molecular mechanisms for the discovery of tumor suppressors and therapeutic options.

### Supplementary Information

**Additional file 1.**

**Additional file 2.**

**Additional file 3.**

**Additional file 4.**

**Additional file 5.**

## Data Availability

All data generated or analyzed during this study are included in this published article and its additional files. The microarray data have been uploaded in NCBI Gene Expression Omnibus (GEO) and the GEO accession number is GSE85716.

## Abbreviations

circRNAs: Circular RNAs
miRNA: MicroRNA
ncRNAs: Non-coding RNAs
NSCLC: Non- small cell lung cancer
qRT-PCR: Real-time quantitative PCR
PIK3: Phosphatidylinositol 3-kinase
RBP: RNA-binding protein
IRES: Internal ribosomal entry site
TNM: Tumor-node metastasis
HBE: Human bronchial epithelial cell line
FBS: Fetal bovine serum
FISH: Fluorescence in situ hybridization
SPF: Specific pathogen-free
PDX: patient-derived xenograft model
