# Supplementary material for: Circadian gene ARNTL initiates circGUCY1A2 transcription to suppress non-small cell lung cancer progression via miR-200c-3p/PTEN signaling
Source: J Exp Clin Cancer Res. 2023 Sep 4;42:229. doi: 10.1186/s13046-023-02791-1 (PMC10478228; doi:10.1186/s13046-023-02791-1)
Supplement: Supplementary file 3 — Additional file 3. [file 13046_2023_2791_MOESM3_ESM.docx]

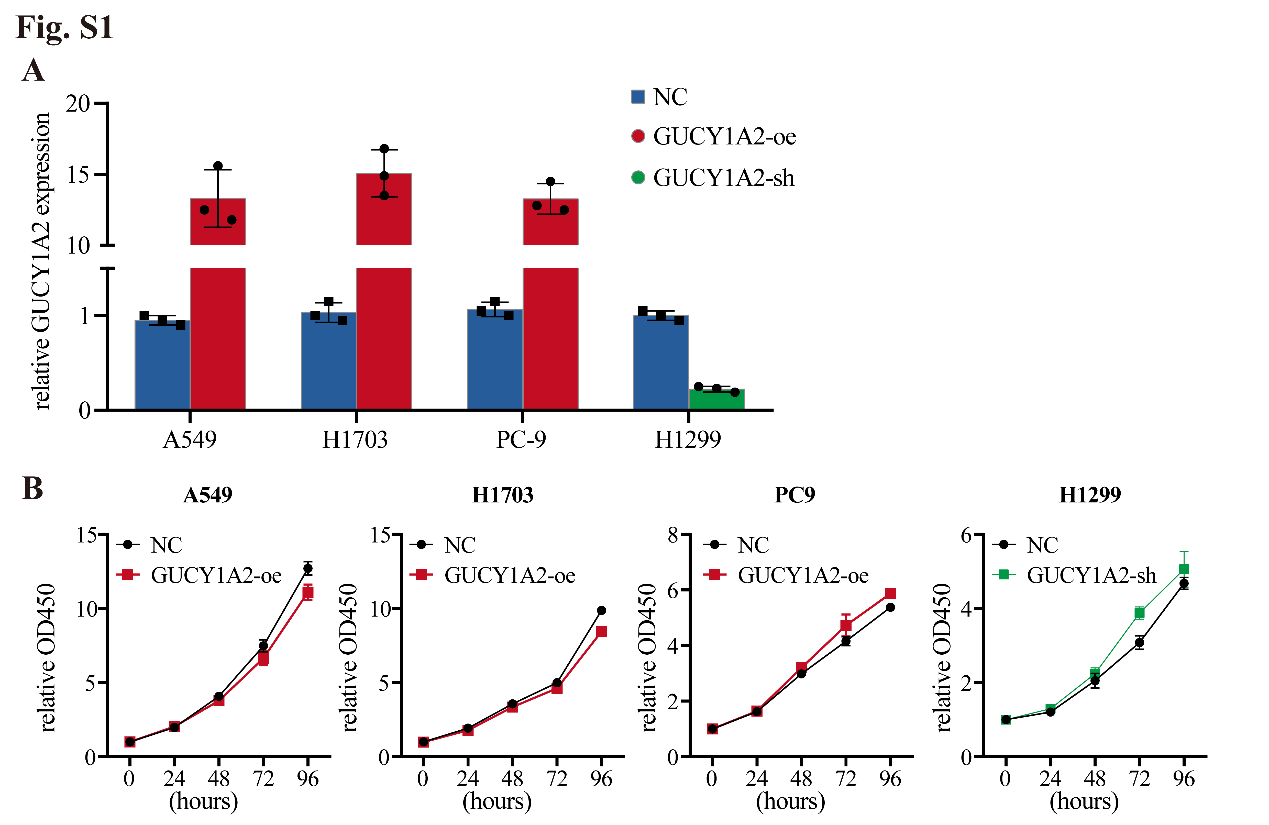


**Figure S1. GUCY1A2 did not significantly affect the proliferation of NSCLC cells. (A) The expressions of mRNA-GUCY1A2 were determined with qPCR in NSCLC cells transfected with negative control or GUCY1A2. (B) Assessment of proliferation of A549, H1703, PC-9 and H1299 cells transfected with negative cotnrol or GUCY1A2 by CCK-8 assay.**


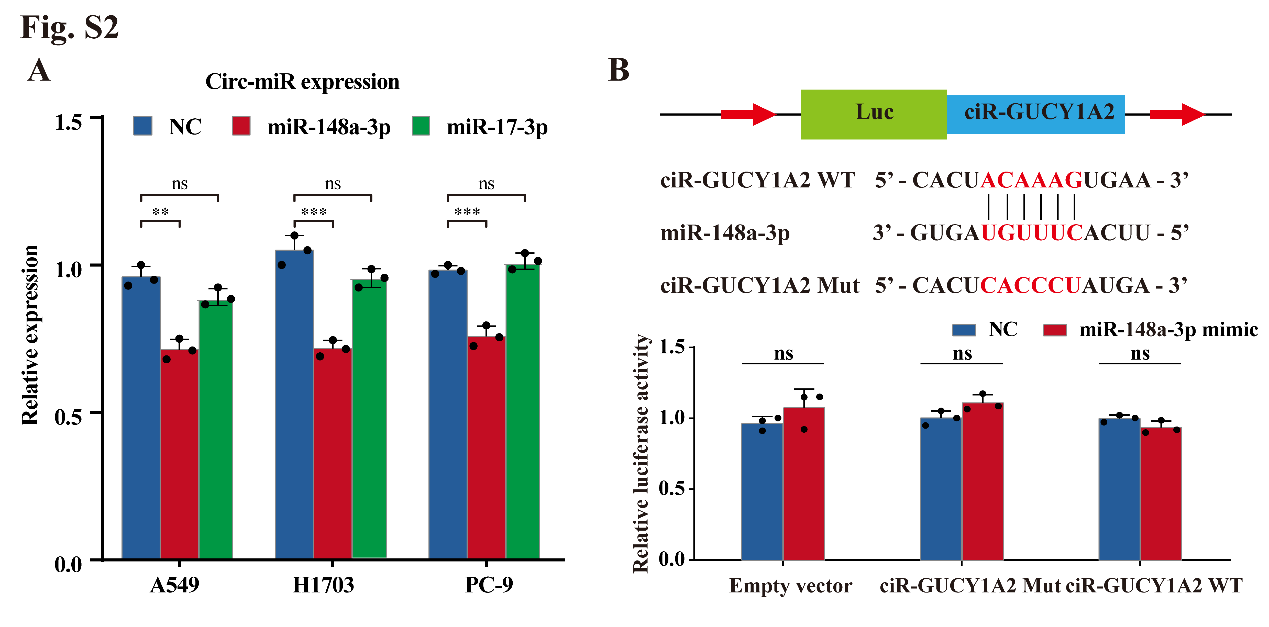


**Figure S2. circGUCY1A2 did not significantly affect the expression of miR-148a-3p and miR-17-3p. (A) The expressions of miR-148a-3p and miR-17-3p were determined with qPCR in NSCLC cells transfected with negative control or circGUCY1A2. (B)** **Schematic of circGUCY1A2 wild-type (WT) and mutant (Mut) luciferase reporter vectors. The relative luciferase activities were analyzed in A549 cells co-transfected with miR-148a-3p mimics or miR-NC and luciferase reporter vectors pLCDH-circGUCY1A2-WT or pLCDH-circGUCY1A2-Mut.**


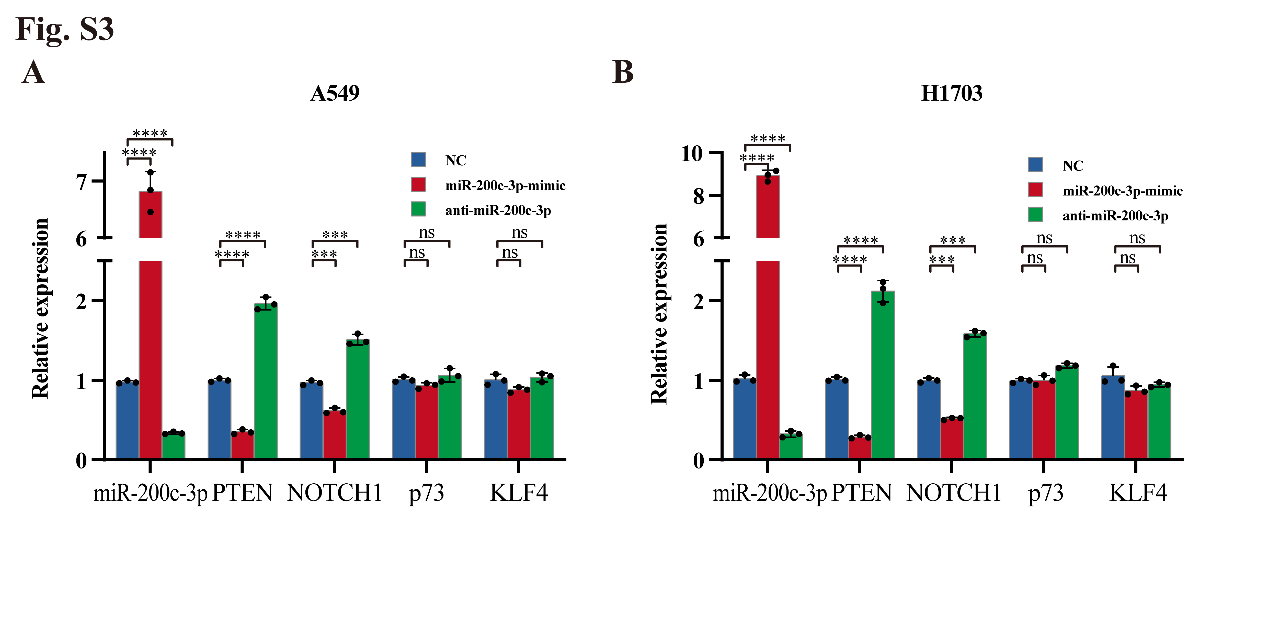


**Figure S3. miR-200c-3p affects the expression of PTEN and NOTCH1 in (A) A549 and (B) H1703.**


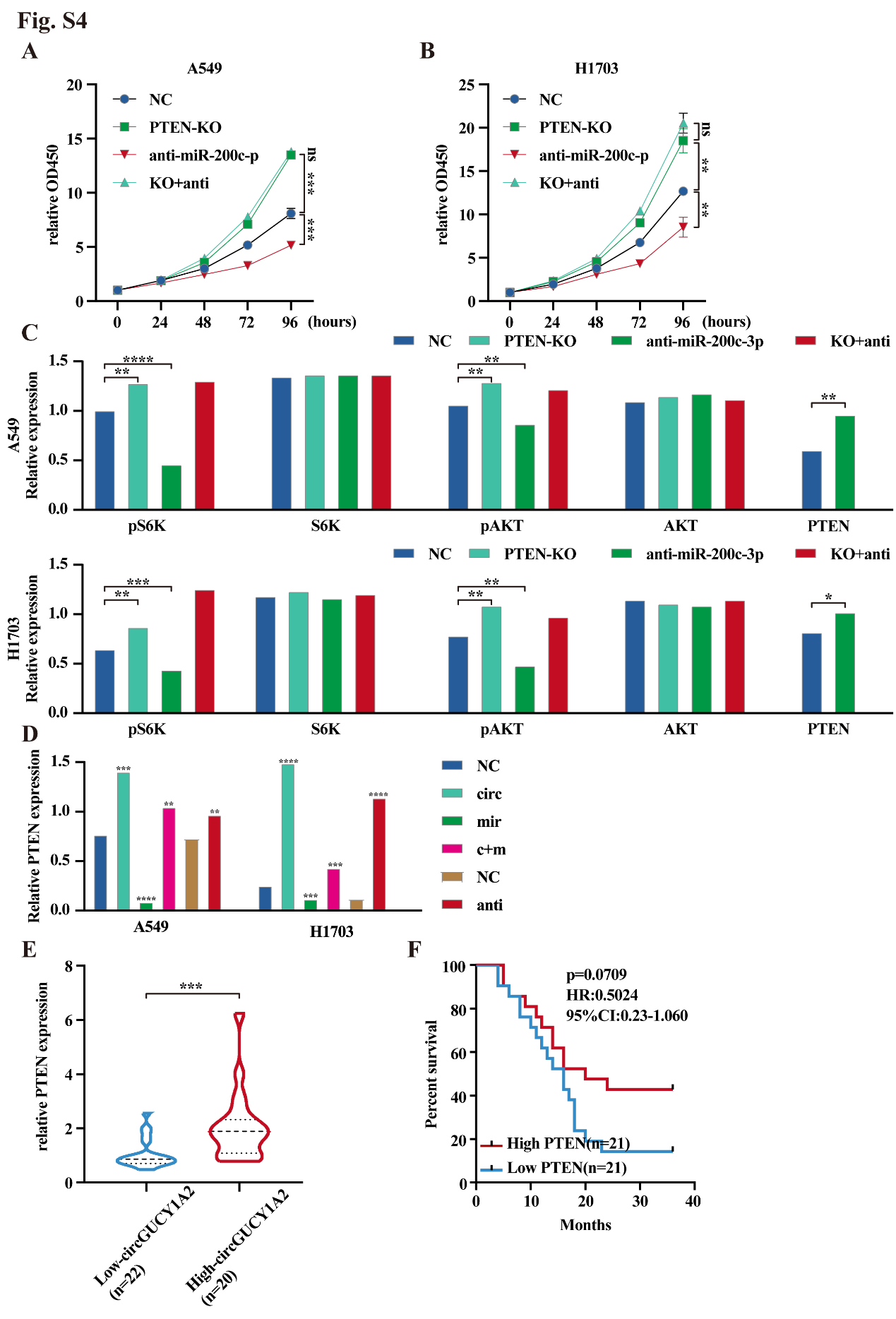


**Figure S4. Inhibition of miR-200c-3p suppresses cell proliferation but is rescued when PTEN is KO. (A) A549 and (B) H1703. (C)** **The grey value analysis of figure 4F. (D) The grey value analysis of figure 4G. (E)** **the expression of PTEN between high- and low- expression of circGUCY1A2 patients. (F) overall survival between high- and low- expression of PTEN patients.**


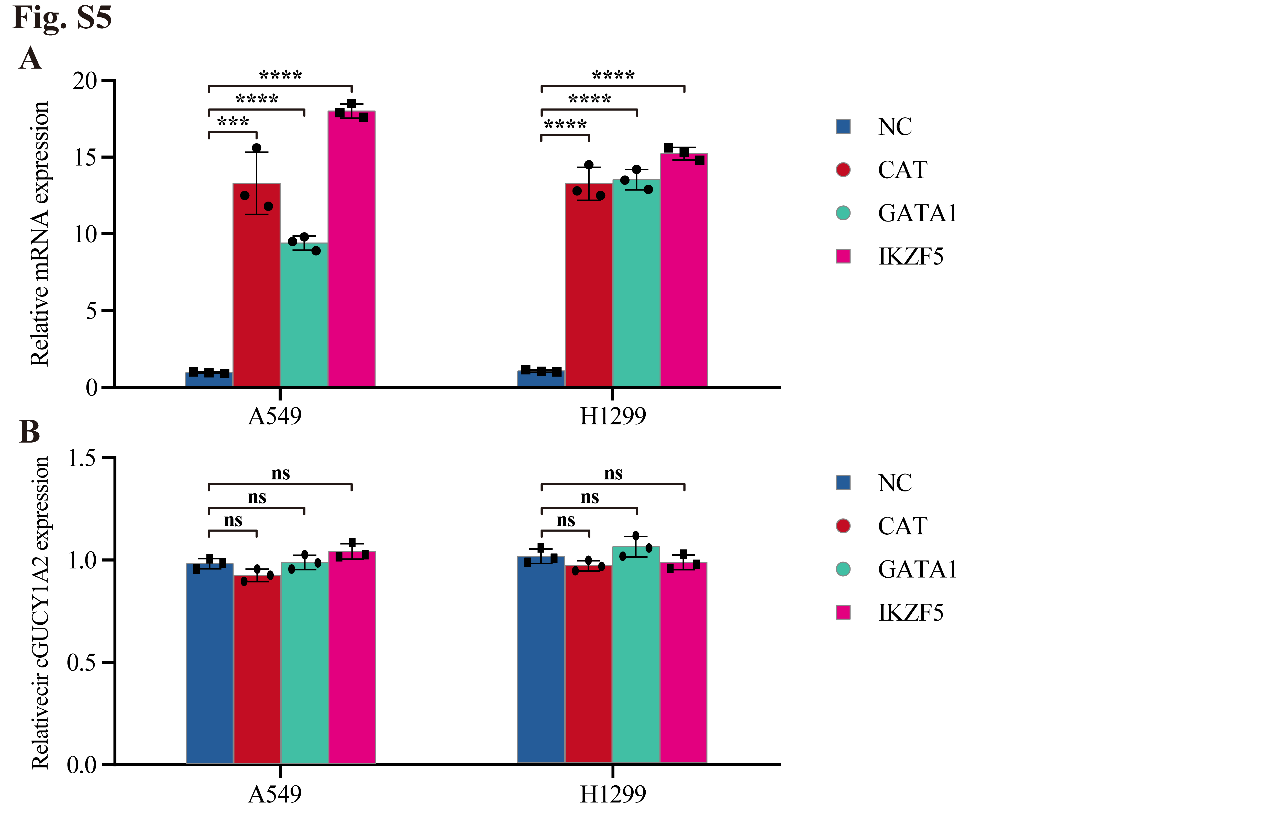


**Figure S5. The effect of overexpression of transcription factors on circGUCY1A2. (A) qRT-PCR verification of transcription factor overexpression, (B) The effect of transcription factor overexpression on circGUCY1A2.**


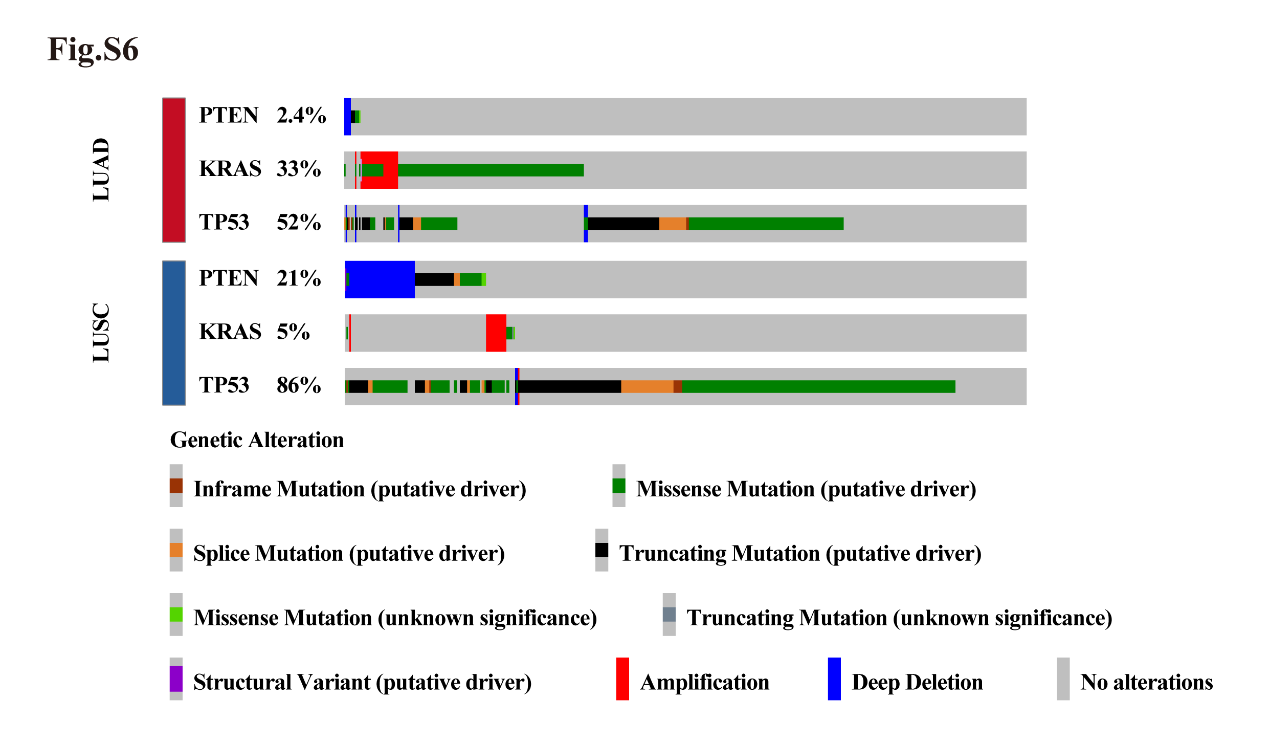


**Figure S6. The frequency of PTEN mutations and deletions of patients from TCGA datasets.**
